# End‐to‐end unsupervised cycle‐consistent fully convolutional network for 3D pelvic CT‐MR deformable registration

**DOI:** 10.1002/acm2.12968

**Published:** 2020-07-13

**Authors:** Yi Guo, Xiangyi Wu, Zhi Wang, Xi Pei, X. George Xu

**Affiliations:** ^1^ Department of Engineering and Applied Physics University of Science and Technology of China Hefei Anhui China; ^2^ Department of Radiology The First Affiliated Hospital of Anhui Medical University of China Hefei Anhui China; ^3^ Nuclear Engineering Program Rensselaer Polytechnic Institute Troy NY USA

**Keywords:** cycle‐consistent, deformable registration, FCN, MR‐CT

## Abstract

**Objective:**

To improve the efficiency of computed tomography (CT)‐magnetic resonance (MR) deformable image registration while ensuring the registration accuracy.

**Methods:**

Two fully convolutional networks (FCNs) for generating spatial deformable grids were proposed using the Cycle‐Consistent method to ensure the deformed image consistency with the reference image data. In all, 74 pelvic cases consisting of both MR and CT images were studied, among which 64 cases were used as training data and 10 cases as the testing data. All training data were standardized and normalized, following simple image preparation to remove the redundant air. Dice coefficients and average surface distance (ASD) were calculated for regions of interest (ROI) of CT‐MR image pairs, before and after the registration. The performance of the proposed method (FCN with Cycle‐Consistent) was compared with that of Elastix software, MIM software, and FCN without cycle‐consistent.

**Results:**

The results show that the proposed method achieved the best performance among the four registration methods tested in terms of registration accuracy and the method was more stable than others in general. In terms of average registration time, Elastix took 64 s, MIM software took 28 s, and the proposed method was found to be significantly faster, taking <0.1 s.

**Conclusion:**

The proposed method not only ensures the accuracy of deformable image registration but also greatly reduces the time required for image registration and improves the efficiency of the registration process. In addition, compared with other deep learning methods, the proposed method is completely unsupervised and end‐to‐end.

## 1 INTRODUCTION

Deformation registration is a process in which one medical image dataset undergoes a series of spatial transformations to match the anatomical structure defined in another medical image dataset.^1^, ^2^ Conventional deformable registration methods include surface‐based methods, point‐based methods, and voxel‐based methods.^3^ The goal of voxel‐based methods is to obtain geometric transformation parameters by calculating metrics between two input image datasets without pre‐extracting features.^4^ However, it is very time‐consuming in iterative calculation of metrics such as mutual information (MI).^5^ Other methods such as intensity‐based feature selection algorithms extract features that correspond well with respect to the intensity; however, they do not necessarily correspond well in regards to the anatomy.^6^, ^7^, ^8^

Recently, many studies have demonstrated the feasibility of deep learning methods for image registration. Cao et al.^9^ proposed an approach based on deep regression networks to predict the deformation field between a pair of image datasets. In other papers,^10^, ^11^ CNN was used to perform fast image registration of three‐dimensional (3D) pulmonary computed tomography (CT) images by combining multiple random transformations to generate a large training set. Rohé et al.^12^ proposed the SVF‐Net architecture using segmented shapes. All the above registration methods need pre‐registration data, contour data, or synthetic data to train neural networks. However, it is difficult to obtain well‐registered clinical medical images and synthetic images are quite different from the actual clinical situation.

To overcome the shortcomings of supervised registration methods, some researchers proposed unsupervised registration methods. Shan et al.^13^ built an end‐to‐end unsupervised learning system with fully convolutional neural networks in which image‐to‐image medical image registration is performed. Hering et al.^14^ presented an unsupervised deep‐learning‐based method in 3D thoracic CT registration using the edge‐based normalized gradient fields distance measure (NGF). Low‐dimensional vectors instead of image pairs were used as input to generate spatial transformation fields in Ref. [15]. Bob et al.^16^ used the deformable image registration network (DIRNet) to register images by directly optimizing a similarity metric between the fixed and the moving image. Balakrishnan et al.^17^ developed a novel registration method that learns a parametrized registration function from a collection of volumes using CNN. Although unsupervised registration methods do not require pre‐registered data and thus have an advantage over supervised registration methods, most unsupervised methods ignore the inherent inverse‐consistent property of transformations between a pair of images.^18^

Generative Adversarial Network (GAN) is a deep learning method, which can make the generated data to have the same distribution as the real data.^19^ To overcome the difficulty of acquiring image pairs in some applications, Zhu and Isola^20^, ^21^ proposed Cycle‐Consistent Adversarial Networks (CycleGAN) to learn a mapping from input to output images without paired training examples. Recently, some studies on GANs for medical image registration have been reported. Mahapatra et al.^5^ used GANs for multimodal medical image registration by adopting novel constraints in the cost function and deformation field reversibility. Fan et al.^22^ proposed an adversarial similarity network to automatically learn the similarity metric for training a deformable registration network. However, these methods still need pre‐registered or predefined aligned images. Another work by Tanner^23^ based on CycleGAN investigated the usefulness of a fully unsupervised MR‐CT image modality synthesis method for deformable image registration of MR and CT images. But this study only used CycleGAN for image synthesis, not for image registration directly. Elmahdy et al^24^ used unsupervised GANs for joint registration in prostate CT radiotherapy; however, their method was not suitable for multi‐modal image registration because they synthesized real samples through artificial deformations which are not useful for multi‐modal image registration. Kim et al^25^ proposed a cycle‐consistent CNN to register multiphase liver CT images, but their method was also not suitable for CT‐MR registration because the loss functions they used could not evaluate the similarity between CT and MR images.

In this paper, we propose a model of using the Cycle‐Consistent method from CycleGAN for 3D CT‐MR deformable registration. This model is end‐to‐end and does not require the ground truth deformations. Our contributions include the following: (a) Using Cycle‐Consistent method in MR‐CT registration to make the deformed image consistent with the reference image, (b) comparing the registration results with and without Cycle‐Consistent, and (c) complete end‐to‐end unsupervised 3D MR‐CT registration network.

## 2 MATERIALS AND METHODS

### 2.A. Deformable image registration framework

**Fig. 1:**
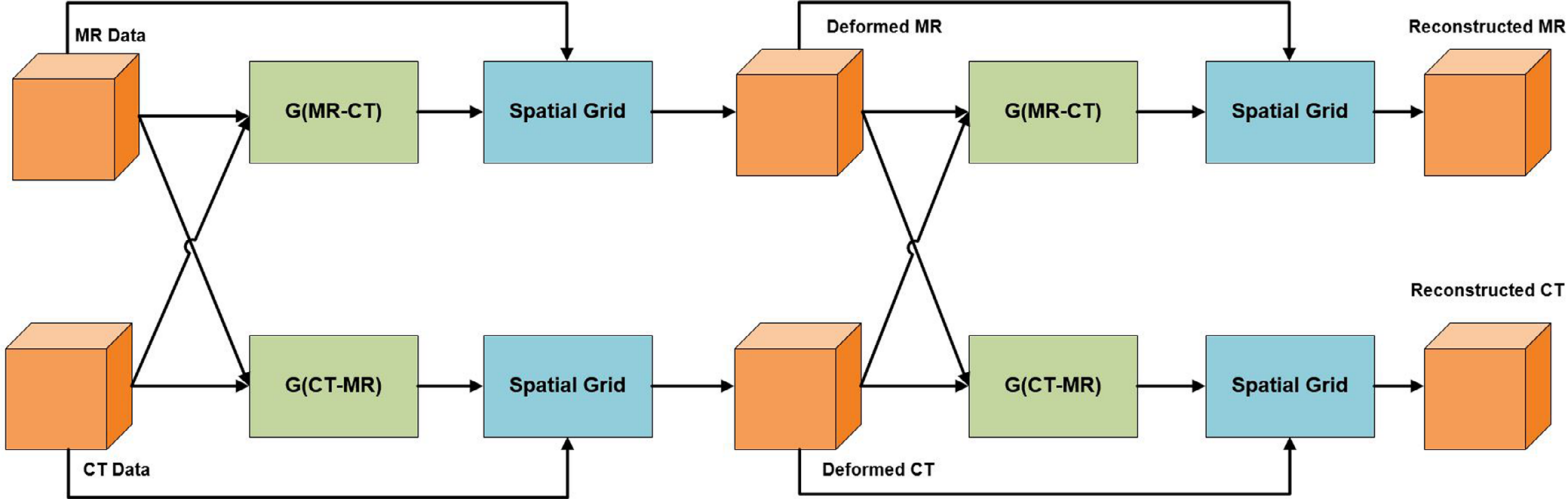
Flow chart of the proposed model. G_MR‐CT_ is a fully convolutional network (FCN) to get the transformation from magnetic resonance (MR) to computed tomography (CT), while G_CT‐MR_ is the opposite. MR and CT are input data and the transform fields are output by two FCNs. The deformed images are used as the input of G_MR‐CT_ and G_CT‐MR_ again and the reconstructed MR and CT are obtained for loss calculation.

The proposed model in this study is Cycle‐Consistent FCNs which is divided into two deformation networks: the G_CT‐MR_ and G_MR‐CT_. G_MR‐CT_ takes MR image as reference image and deforms CT, G_CT‐MR_ takes CT image as reference image and deforms MR. The flow chart of the model framework is shown in Fig. 1, the deformation network first receives multimodal image pairs (CT and MR) and outputs the deformed transformation. Then the moving images are deformed to get the deformed images. After that, the deformed image pairs are input into the deformation network again to obtain the reconstructed transformation and reconstructed image pairs.

### 2.B. Patient data preprocessing

In all, 74 pelvic cases including CT images and MR images are used as datasets. We standardize all image data to make the distribution range of pixel values of all images consistent, and resample them to a resolution of 1 1 5 mm^3^. To reduce the size of input data and highlight the regions to be registered, each image is cropped to 400 400 voxels so that the redundant air areas are removed. Due to the limitation of compute video memory size, it is necessary to resample the training data to 200 × 200 24 voxels before the training process. Rigid registration is carried out for all the cases using 3Dslicer software^26^ because it can reduce the difficulty of the neural network training. Finally, we normalize the image data and map all the image pixel values to the range of (−1, 1).

### 2.C. Deformation network and Loss function

**Fig. 2:**
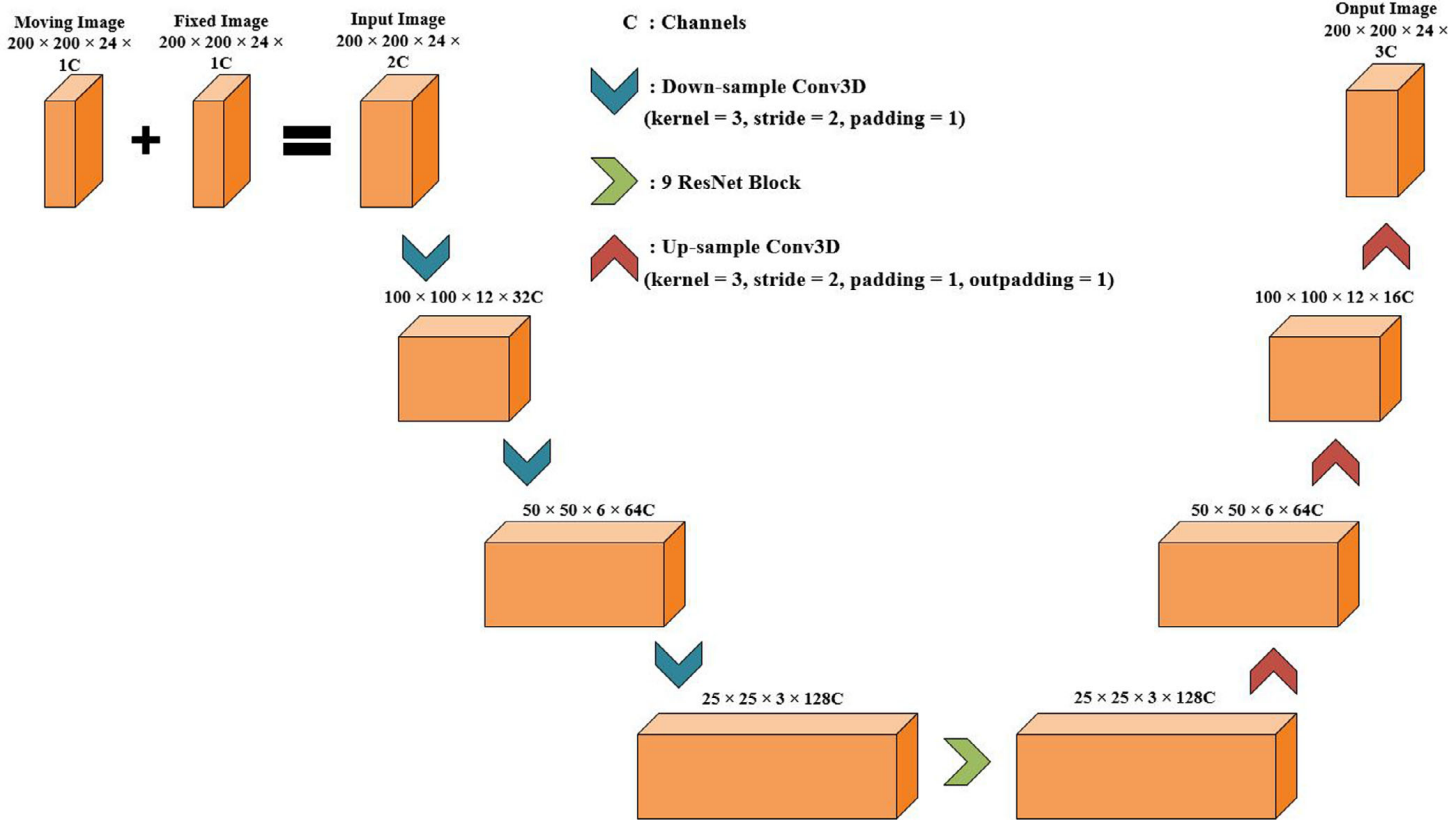
The structure of the fully convolutional network (FCN). Moving image [magnetic resonance (MR)/computed tomography (CT)] and fixed image (CT/MR) are combined into a two channel image as the input of the network. After three down‐sampling convolution layers, one ResNet block and three up‐sampling layers, the input data finally become a deformation field with the same size as the input image.

The objective of the deformation network is to obtain the spatial deformed transformation according to the input image pairs. We use spatial deformation field to describe the process of deformation registration. The structure of the deformation network is shown in Fig. 2, the network expects a pair of multimodal images with size 200 × 200 24 voxels. Three 3 3 3 convolution layers (with two‐stride and one‐padding) then down‐sample the input images and the activation function is ReLU. To increase the depth of the network and make it easier to optimize, nine ResNet Blocks are used.^27^ After the down‐sampling layers and ResNet Blocks, the images need to be up‐sampled to get the spatial transformation grid finally. Three 3 3 3 convolution layers up‐sample the data and each convolution layer has different parameters including stride and padding.

The loss function of deformation network consists of three parts. ① Content loss L_cont_, which can ensure that the deformed image has the desired characteristics. ② Regularization loss L_reg_, the objective of which is to smooth the deformation field. ③ Cycle loss L_cyc_, which can ensure the inherent inverse‐consistent property of transformations between a pair of images.

#### 2.C.1. Content loss

The most common metric for multimodal image registration is MI. However, MI metric ignores the spatial neighborhood of a particular voxel within one image and consequently, which causes the decrease in registration accuracy in deformable registration.^28^ To solve this problem, we use a metric called modality‐ independent neighborhood descriptor (MIND) to perform deformation registration on CT‐MR images.^28^

The MIND feature extracts distinctive image structure by comparing each patch with all its neighbors in a non‐local region.^29^ Formula (1) shows the MIND feature extraction function, where n is a constant to normalize the function R indicate the spatial search region.

(1) $$MIND\left(I,x,r\right)=\frac{1}{n}\exp\left(‐\frac{D\left(I,x,x+r\right)}{V\left(I,x\right)}\right),r\in R$$

D represents the L2 distance between two image blocks in image I centered on voxel x and voxel x + r, respectively. The detailed function of D is shown in Formula (2), where *P* denotes image Patch and we set the patch size to 5 × 5 during model training.

(2) $$D\left(I,x,r\right)=\sum_{p\in P}{\left(I\left(x+p\right)‐I\left(x+r+p\right)\right)}^{2}$$

V is a variance estimate on voxel x and its function is shown in Formula (3), where N is the 3 neighborhood of the voxel x.

(3) $$V\left(I,x\right)=\frac{1}{3}\sum_{n\in N}D\left(I,x,x+n\right)$$

We can calculate the content loss between CT image and MR image based on MIND feature extraction function. As shown in Formula (4), where N represents the number of image voxels, R is the spatial search region and we set the region size to 7 × 7 during model training.

(4) $$L_{cont}=\frac{1}{N\left|R\right|}\sum_{x\in N}\sum_{r\in R}\left|MIND\left(CT,x,r\right)‐MIND\left(MR,x,r\right)\right|$$

#### 2.C.2. Regularization loss

To prevent unreasonable deformation, we add regularization loss to make the deformation grid smoother. L2 regularization is used to evaluate the deformation field and its function is shown in Formula (5).

(5) $$L_{\mathrm{reg}}=∥D_{f}|{|}_{2}$$

where D_f_ denotes the deformation grid.

#### 2.C.3. Cycle loss

Cycle loss enables the deformed image to be deformed back to the original image. In addition, cycle loss can prevent some excessive deformation and make the model easier to converge. Formula (6) shows the function of Cycle loss, where G means the network generating deformation field, I_m_ is the moving image, and I_f_ is the fixed image. G(I_m_, I_f_) denotes that I_m_ is deformed to be similar to I_f_ and G(I_f_, I_m_) was the opposite.

(6) $$L_{cyc}=||G\left(G\left(I_{m},I_{f}\right),G\left(I_{f},I_{m}\right)\right)‐I_{m}|{|}_{1}$$

#### 2.C.4. Total loss for model

The total loss L_G_ of the model we proposed is composed of all the above loss and we set coefficients for different loss as shown in Formulas (7), (8), and (9). L_MR‐CT_ represents that MR image is the moving image and L_CT‐MR_ means CT image is the moving image. λ_1_, λ_2_, and λ_3_ are constants to adjust the proportion of different loss in the total loss. We set λ_1_ to 5, λ_2_ to 1, λ_3_ to 1 during model training.

(7) $$L_{MR‐CT}=\lambda_{1}L_{cont}\left(MR,CT\right)+\lambda_{2}L_{reg}\left(MR,CT\right)+\lambda_{3}L_{cyc}\left(MR,CT\right)$$

(8) $$L_{CT‐MR}=\lambda_{1}L_{cont}\left(CT,MR\right)+\lambda_{2}L_{reg}\left(CT,MR\right)+\lambda_{3}L_{cyc}\left(CT,MR\right)$$

(9) $$L_{G}=L_{MR‐CT}+L_{CT‐MR}$$

## 3 RESULTS AND DISCUSSION

Python and Pytorch was used to implement our model, and Adam was used as the optimizer. We compared the results of the proposed method with those of the registration software Elastix, MIM software, and FCN without Cycle‐Consistent in terms of registration accuracy and registration speed.^21^, ^22^, ^30^ The registration parameters in Elastix: interpolator is “BSplineInterpolator,” Optimizer is "AdaptiveStochasticGradientDescent," Transform is "BSplineTransform," Metric is "AdvancedMattesMutualInformation," and MaximumNumberOfIterations is 5000. Training and testing were performed on a computer with Intel i7‐8700 K CPU, 16GB Memory, NVIDIA GeForce GTX 1070 Ti GPU, and 8 GB Video Memory.

For evaluation purposes, the region of interest (ROI) of all cases had been outlined in advance, including rectum and bladder. We calculated the Dice coefficient and ASD of ROIs before registration and after registration. Tables 1 and 2 show the Dice values and ASD of rectum and bladder in ten test cases before registration and after registration, and the * indicates the best Dice value or best ASD in the corresponding test case. We can see that, FCN with Cycle‐Consistent performed best among the four registration methods tested and it was more stable in general. Although the other three methods sometimes showed good registration results, they are not stable enough and easy to get unreasonable deformation. In terms of registration time, the Elastix method took the longest time and the FCN with or without Cycle‐Consistent methods took <0.1 s per case. For the MIM method, the user needs to adjust the registration image manually, which will take a lot of time and require the user to have experience.

**Table 1:** Dice values, average surface distance (ASD), and registration time of Rectum in pelvic cases before registration, after registration.

Rectum	Before registration		Elastix		MIM		FCN with cycle‐consistent (our method)		FCN without cycle‐consistent	
	Dice	ASD (mm)	Dice	ASD (mm)	Dice	ASD (mm)	Dice	ASD (mm)	Dice	ASD (mm)
Case1	0.26	13.26	0.71	4.58	0.68	5.72	0.71	4.30	0.76*	3.86*
Case2	0.42	10.56	0.82*	2.59*	0.62	5.17	0.70	3.18	0.75	3.88
Case3	0.48	15.04	0.81*	3.82	0.76	4.07	0.75	3.51*	0.75	4.35
Case4	0.59	10.43	0.66	6.58	0.72	3.64	0.87*	1.54*	0.86	1.70
Case5	0.54	14.57	0.82	3.21	0.84*	2.63*	0.77	3.55	0.79	3.67
Case6	0.10	18.95	0.69	2.22	0.53	3.07	0.75*	1.58*	0.44	3.68
Case7	0.46	10.11	0.88	2.77	0.67	5.94	0.85	1.98*	0.89*	2.18
Case8	0.53	14.72	0.60	5.99	0.69	4.44	0.91*	1.15*	0.74	3.84
Case9	0.60	21.18	0.82	4.58	0.89*	2.96	0.89*	2.82*	0.88	3.06
Case10	0.35	13.03	0.80	3.63	0.88*	2.26*	0.83	2.94	0.75	4.08
Average result	0.43	14.19	0.76	4.00	0.73	3.99	0.80	2.66	0.76	3.43
Standard deviation	0.16	3.63	0.09	1.44	0.12	1.30	0.08	1.04	0.13	0.86
Average time	/		64 s		28 s		<0.1 s		<0.1 s	

The * indicates the best Dice value or best ASD in the corresponding test case.

**Table 2:** Dice values, average surface distance (ASD), and registration time of Bladder in pelvic cases before registration, after registration.

Bladder	Before registration		Elastix		MIM		FCN with cycle‐consistent (our method)		FCN without cycle‐consistent	
	Dice	ASD (mm)	Dice	ASD (mm)	Dice	ASD (mm)	Dice	ASD (mm)	Dice	ASD (mm)
Case1	0.54	13.74	0.77	6.78	0.77	6.88	0.87*	3.91*	0.86	3.94
Case2	0.66	14.45	0.82	5.97	0.69	10.25	0.86*	5.15*	0.81	6.60
Case3	0.75	14.35	0.91	1.95	0.86	3.23	0.92*	1.75*	0.91	2.23
Case4	0.33	21.84	0.80	4.37	0.86*	2.50*	0.83	3.39	0.80	4.53
Case5	0.76	15.21	0.84	4.65	0.89*	2.95	0.89*	2.61*	0.89*	2.88
Case6	0.50	17.63	0.89*	3.73*	0.82	6.23	0.86	3.73*	0.83	5.84
Case7	0.63	9.96	0.79	6.28	0.87*	3.59	0.87*	3.02*	0.87*	3.31
Case8	0.63	16.05	0.89*	2.53*	0.79	5.14	0.84	3.07	0.83	3.68
Case9	0.40	15.29	0.74	6.57	0.82	4.64	0.82	4.51	0.85*	3.50*
Case10	0.53	14.84	0.83*	5.66	0.80	5.98	0.83*	5.06*	0.73	8.33
Average result	0.57	15.34	0.83	4.85	0.82	5.14	0.86	3.62	0.84	4.48
Standard deviation	0.14	3.01	0.06	1.70	0.06	2.34	0.03	1.08	0.05	1.89
Average time	/		64 s		28 s		<0.1 s		<0.1 s	

The * indicates the best Dice value or best ASD in the corresponding test case.

By comparing the registration results of bladder and rectum, we can find that the average Dice coefficients of bladder are higher than that of rectum in all four registration methods. This may be because the contour of bladder is larger than that of rectum, and the three‐dimensional deformation registration often brings complex deformation field, so in order to meet the overall alignment between the image pairs, the local deformation will not be accurate enough. Therefore, when the ROI is not obvious enough in the image pairs, the smaller the contour of the ROI, the lower the accuracy of registration. In addition, it can be observed that in 6 of the 10 test cases, our method achieves the best Dice coefficient of bladder. However, only in 4 of the 10 test cases, the method proposed get the best Dice coefficient of rectum. It can be inferred that our method pays more attention to the whole alignment of image pairs in the training process, but is less sensitive to the small organs. On the other hand, our method performs the best ASD for both rectum and bladder in ten test cases. Although the results show that the cases with high Dice coefficient also have high ASD, our method still get high ASD score in some cases with low Dice coefficient. It can be inferred that the shapes of deformed ROI contours obtained by our method are closer to the shapes of target contours. Taken together, our method shows satisfactory registration results compared with the existing methods.

**Fig. 3:**
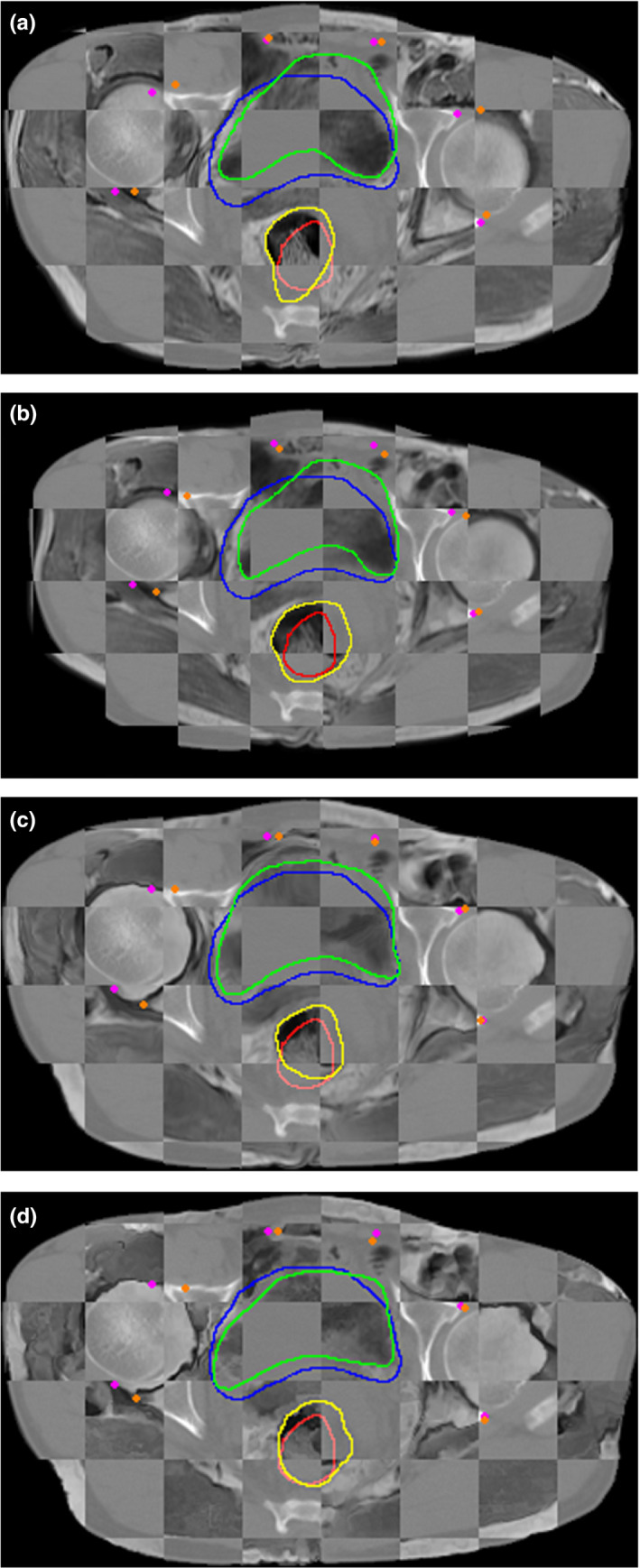
Checkboard fusion images of computed tomography (CT) and magnetic resonance (MR) after registration with different methods. (a) the fusion image using Elastix; (b) the fusion image using MIM; (c) the fusion image using fully convolutional network (FCN) with Cycle‐Consistent; (d) the fusion image using FCN without Cycle‐Consistent. The red, blue, yellow, and green contours represent the rectum of fixed image, the bladder of fixed image, the rectum of deformed moving image, and the bladder of deformed moving image. The purple and orange points represent the corresponding points of CT and deformed MR.

**Fig. 4:**
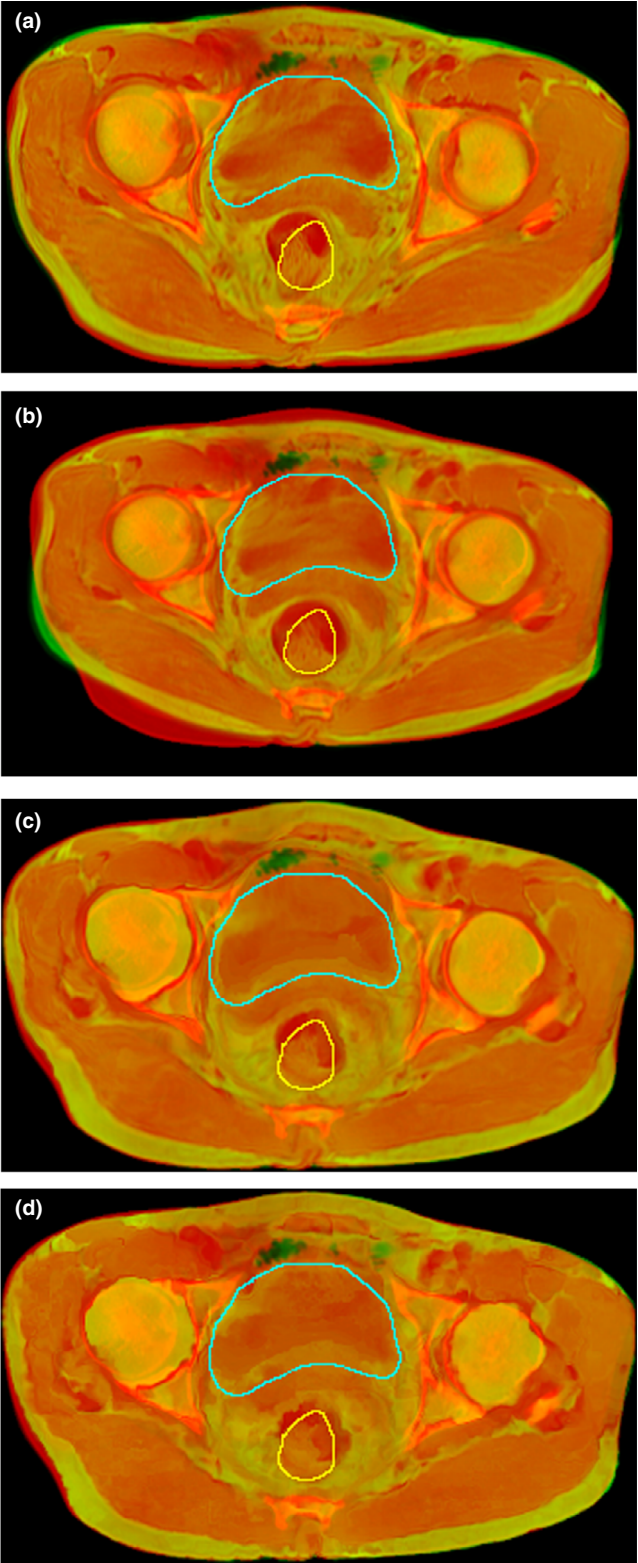
Color‐coded fusion images of computed tomography and magnetic resonance after registration with different methods. (a) the fusion image using Elastix; (b) the fusion image using MIM; (c) the fusion image using fully convolutional network (FCN) with cycle‐consistent; and (d) the fusion image using FCN without cycle‐consistent. The yellow, green contours represent the rectum of fixed image, the bladder of fixed image.

Figures 3 and 4 show the checkboard fusion images the color‐coded fusion images of CT and MR and the contours of the ROIs. It can be seen that the result of Elastix method was not satisfactory for the caput femoris and ROIs although it successfully aligned the contour of skin and muscles. The result of MIM method did better in ROIs registration than Elastix, but it was not good enough in the alignment of skin contour. This is because in the process of registration using MIM software, users need to manually set the reference point and registration box to improve the local registration accuracy, which makes the registration results of MIM software can achieve high accuracy in the local area, but slightly lacking in the whole. The result of FCN with or without Cycle‐Consistent performed well in aligning ROIs. However, the deformation fields of FCN without Cycle‐Consistent were not smooth enough and prone to unreasonable deformation. To further evaluate the registration accuracy surround ROIs, several corresponding points (such as the points on the edges of bones, etc.) were added in CT and MR to calculate the TRE (Target registration Error). The purple and orange points in Fig. 3 represent the corresponding points in CT and deformed MR using different methods. The average TRE values of Elastix, MIM software, the proposed method, and FCN without Cycle‐Consistent were 7.60, 8.15, 7.04, and 7.53 mm, respectively. Although the number of corresponding points is small, the result still reflects the proposed method can improve the registration accuracy to a certain extent.

To explore the influence of the parameters of the metric MIND on MR‐CT registration, we changed the patch size, the region size, and the neighborhood size of a voxel in Formulas (2), (3), and (4), but did not get better or worse registration result.

## 4 CONCLUSIONS

Iterative calculation is the most common method in medical image registration, but it is relatively time‐consuming. In this paper, a 3D MR‐CT image deformation registration method based on Cycle‐Consistent FCN is proposed. Compared with other existing image registration networks, this model was end‐to‐end and completely unsupervised. ResNet Block was used to increase the depth of the network. The results show that the proposed model in this study can accurately register multi‐modal medical images and greatly improve the registration speed. In future research, we plan to apply this model to other medical image registration progress (different modalities or different body parts). At the same time, further clinical validation and application are also under way.

## CONFLICT OF INTEREST

There are no conflict of interest.
